# Veteran Engagement in Health Services Research: a Conceptual Model

**DOI:** 10.1007/s11606-021-07309-z

**Published:** 2022-03-29

**Authors:** Sara J. Knight, Jeffrey P. Haibach, Alison B. Hamilton, Jeff Whittle, Sarah S. Ono, Jorie Butler, Mark Flower, Carolyn D. Ray, Mary Jo Pugh, Susan L. Zickmund

**Affiliations:** 1 Informatics, Decision Enhancement, and Analytic Sciences (IDEAS) Center of Innovation, Research and Development Service, VA Salt Lake City Healthcare System, 500 Foothill Drive, Salt Lake City, UT USA; 2 Division of Epidemiology, Department of Internal Medicine, University of Utah, Salt Lake City, UT USA; 3 Veteran Consulting and Research, Eastern Region, USA; 4 US Department of Veterans Affairs, Washington, DC USA; 5 Center for the Study of Healthcare Innovation, Implementation & Policy, VA Greater Los Angeles Healthcare System, Los Angeles, CA USA; 6 Department of Psychiatry and Biobehavioral Sciences, University of California Los Angeles, Los Angeles, CA USA; 7 Clement J Zablocki VA Medical Center, Medical College of Wisconsin, Center for Advancing Population Science, Milwaukee, WI USA; 8 Department of Medicine, Clement J Zablocki VA Medical Center, Milwaukee, WI USA; 9 Center to Improve Veteran Involvement in Care, VA Portland Healthcare System, Portland, OR USA; 10 Department of Family Medicine, Oregon Health & Science University, Portland, OR USA; 11 Geriatric Research Education and Clinical Center, VA Salt Lake City Healthcare System, Salt Lake City, UT USA; 12 Veteran Peer Services, Mental Health America of Wisconsin, Milwaukee, WI USA; 13 Birmingham VA Medical Center, Birmingham, AL USA

**Keywords:** Veteran, patient engagement, stakeholder engagement, healthcare system, conceptual model

## Abstract

With 20 million living veterans and millions more immediate family members, and approximately 9 million veterans enrolled in the nationally networked VA healthcare system, representing the interests and needs of veterans in this complex community is a substantial endeavor. Based on the importance of engaging Veterans in research, the VA Health Services Research and Development (HSR&D) Service convened a Working Group of VA researchers and Veterans to conduct a review of patient engagement models and develop recommendations for an approach to engage Veterans in health research that would incorporate their unique lived experiences and interests, and their perspectives on research priorities. The Working Group considered the specific context for Veteran engagement in research that includes other VA stakeholders from the operational and clinical leadership of the VA Health Administration (VHA). The resulting model identifies the range of potential stakeholders and three domains of relevant constructs—processes expected to facilitate Veteran engagement in research with other stakeholders, individual stakeholder and external factors, and outcomes. The expectation is that Veteran engagement will benefit research to policy and practice translation, including increasing the transparency of research and producing knowledge that is readily accepted and implemented in healthcare.

With 20 million living Veterans and millions more immediate family members, and approximately 9 million Veterans enrolled in the national Veterans Health Administration (VHA), representing the perspectives, interests, and needs of Veterans in a complex health system is a substantial endeavor.^1^ The Department of Veterans Affairs (VA) had engaged stakeholders in its national research inititives, including Veterans, their family members, and VHA clinicians and executives,^2–4^ but did not emphasize the engagement of Veterans widely in individual research projects or research centers throughout the nation. In 2010, leaders in the VA Office of Research and Development and its Health Services Research and Development (HSR&D) Service recognized the potential for stakeholder engagement to accelerate the translaton of VA research findings to improvements in medical care and health policy and began to encourage its research centers and researchers to engage national and local leaders and decision-makers (e.g., directors, executives) in the Veterans Health Administration (VHA). The major goal of engaging key decision-makers as stakeholders at the research center and individual level was to increase the relevance of VA research to the VA healthcare delivery system and to the nation.^5^

The effort to engage VA healthcare system leaders was fully underway when the national VA HSR&D Service saw that Veterans were not fully integrated into the earlier stakeholder engagement model and activities.^6,7^ Consideration of the unique context for Veteran engagement in VA research alongside stakeholders serving in executive positions in the VHA contributed to the creation of a working group tasked with focusing on how to best incorporate Veterans’ perspectives in VA research. A Conceptual Model Subgroup was convened to conduct a review of existing conceptual models of patient engagement and to select or develop a model to inform how to best engage Veterans in research where multiple stakeholders were already embedded. A unique Veteran model informing decisions about Veteran engagement would provide a strong foundation for evaluation and research. In this paper, we describe the Conceptual Model Subgroup and development of a Veteran-centric conceptual model.

MODEL DEVELOPMENT

The Conceptual Model Subgroup of the Veterans Engagement Working Group included VA social scientists and health service researchers (JB, AH, SK, SO, JW, SZ) and Veterans (MF, CR). VA scientists were selected based on their expertise in stakeholder engagement and experience in qualitative data analysis. Veterans were selected based on prior service, expertise in patient advocacy, and service to Veterans through the VA. All subgroup members were investigators or stakeholders in VA health service research programs. To accomplish the aims of the subgroup, the members would (1) identify critical elements important to integrating Veterans’ perspectives in VA health service research and (2) develop a preliminary conceptual framework for considering research questions on the mechanisms and outcomes of Veteran engagement.^6,7^

The subgroup used a three-step iterative process: (1) review conceptual models and frameworks including those developed outside the VA and others developed or adapted in the VA and select models that include the elements needed to support rigorous research on engagement mechanisms and outcomes, (2) qualitatively compare selected frameworks and models from previous literature and select a model or constructs and hypothesized relationships from several models for use in a Veteran engagement model, and (3) through deliberation and consensus building incorporate new constructs and linkages among the constructs relevant to Veterans and the VA research experience.

Conceptual frameworks that informed the early dialogue on the Veteran model included those developed by the Patient Centered Outcomes Research Institute,^8,9^ and Clinical and Translational Research Award (CTSA) Initiative.^10^ The subcommittee examined the context where each prior model was intended to be used, engagement of multiple stakeholders, and relevance of proposed processes, influencing factors, and outcomes to the stated goals of Veteran engagement in health services research. They evaluated and considered model strengths (e.g., presence of constructs to support rigorous research on Veteran engagement), weaknesses (e.g., lack of linkage among model elements), and adaptability for VA research (e.g., health delivery model and stakeholders not relevant to VA research).

In step one, the subgroup reviewed ten patient engagement models developed outside the VA and based on principles and strategies from Comparative Effectiveness Research (CER), Implementation Science (IS), and Community-Based Participatory Research.^8,11–19^ One of these models, Isler and Corbie-Smith, had been adapted for Veteran engagement.^15^ After discussion, the subgroup members decided to exclude seven models that focused exclusively on values and principles associated with engagement, simple processes, and stages of engagement. Models were retained if they incorporated domains and constructs that identified stakeholder and environmental characteristics, facilitating factors, outcomes, and other elements depicting how engagement works. Three of the ten models were retained.^8,14,15^ All three included outcome domains, two included domains for predisposing researcher or participant constructs, and two included contextual environmental and organizational factors. The VA adaptation of the Isler and Corbie-Smith model was the only model retained that included multiple stakeholders.^15^

In step two, the subgroup discussed and evaluated constructs from the retained models for use with Veterans. The subgroup reached a consensus that, while no one model had the elements to guide rigorous research, a Veteran engagement model could be developed by selecting elements from the three models, adapting the elements for Veterans, and incorporating the elements in a single model. The domains identified to consider for inclusion in the Veteran model were stakeholder factors, facilitators, organizational context, and outcomes.

The subgroup members considered in step three how the elements of the three models could be adapted to represent the unique characteristics of the VA setting and the multiple-stakeholder-engaged research that the Veterans would be joining as new stakeholders. Each element was adapted to incorporate constructs specific to the VA and Veterans. While the group had previously excluded simple process models of engagement (e.g., awareness, support, change), the subgroup reasoned that adaptive, collaborative, and generative interpersonal processes would be important for Veterans and other VA stakeholders to be able to work together and make substantive contributions to research. Several iterative models were developed and revised with discussion to integrate the processes, improve content and face validity, internal consistency, and parsimony.

The final model is presented in Figure 1. Veteran, researcher, health professional, organizational decision-maker stakeholders are shown in a circle to represent the breadth of interests and perspectives that would be expected in a multi-stakeholder effort. Several other stakeholders are shown below the circle to illustrate the possibility of expansion with the addition of stakeholders such as family members. In the center, group processes necessary for productive collaboration among stakeholders are shown (see Table 1 for examples of interpersonal activities shown to illustrate adaptive, collaborative, and generative processes). Construct domains (partner factors, facilitators, organizational context) and four types of outcomes are shown (mutually reinforcing experiences and research-related, health system–related, and long-term health outcomes). Linkages among the domains of relevant constructs are shown to illustrate possible relationships among the elements.

**Figure 1:**
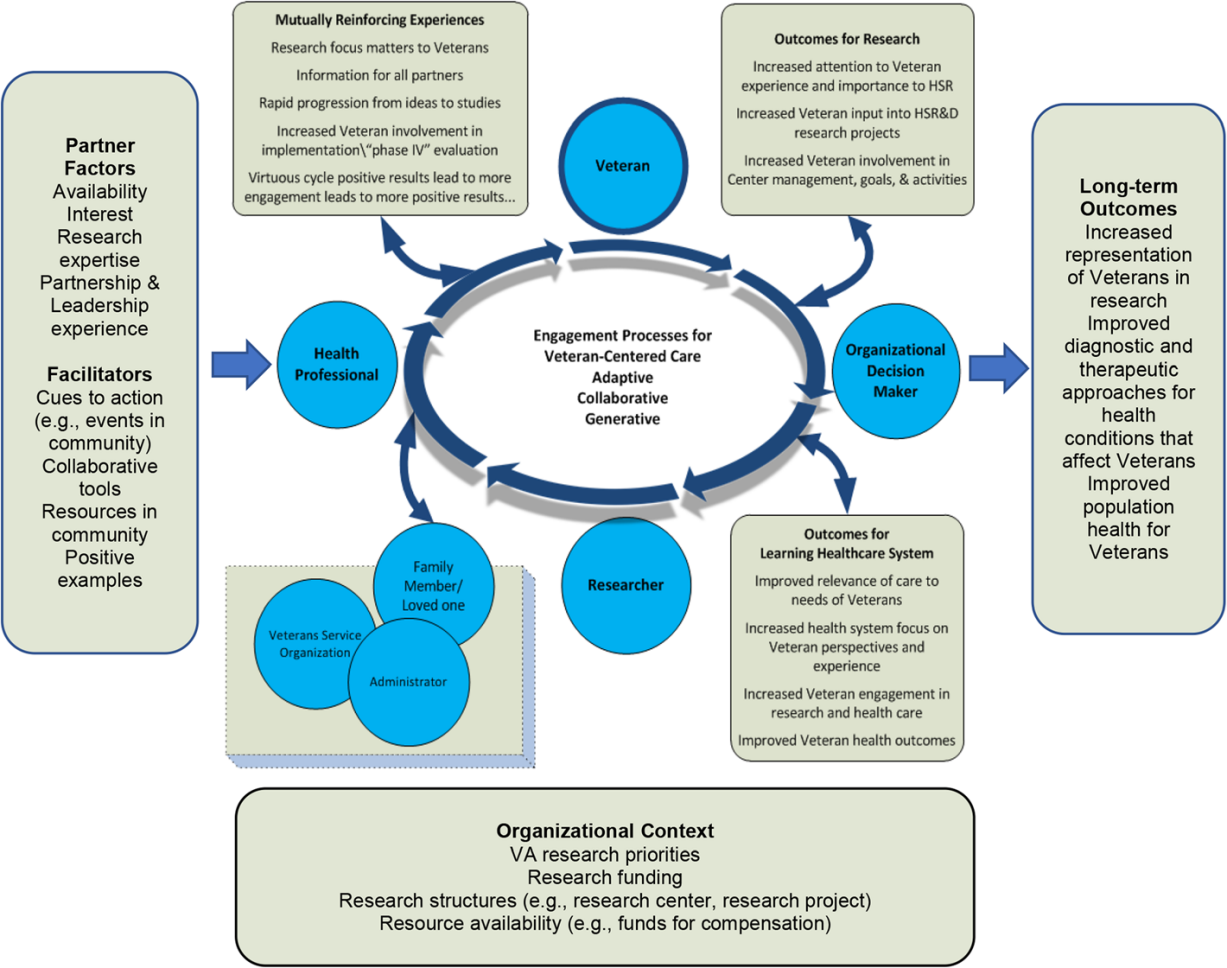
Veteran engagement in research: a conceptual model.

**Table 1:** Veteran Engagement Process Examples

Collaborative	
• Promoting awareness through exchange of information and ideas	
• Establishing and maintaining mutual trust and transparency	
• Learning together as veterans, researchers, and other stakeholders share their knowledge, experience, and insight with one another as partners	
• Communicating effectively	
• Consulting, respectful pragmatism, and analytical discussion	
• Cooperating to achieve project and broader objectives	
• Consensus building to focus the effort (though not everyone has to agree)	
Adaptive	
• Individuals and the group adjusting to the specific partnered dynamic, projects, and tasks	
• Facilitating and mediating with formal or informal moderators/facilitators/coordinators	
• Balancing individual vs group differences or interests, research vs operations	
• Promoting connections and interconnectedness while minimizing fragmentation or cliques	
• Managing projects	
Generative	
• Building the team for a given project and broader objectives	
• Brainstorming, discussions, and ongoing dialogue	
• Developing research questions and hypotheses	
• Planning and development of projects and tasks	
• Conducting and participating in productive meetings	
• Developing capacity	
• Writing (e.g., proposals, funding applications, presentations, reports, publications, brochures)	
• Disseminating—driving progress, quality improvement, and evidence-based change to clinical care and systems	

## DISCUSSION

We propose a conceptual framework for Veteran engagement that addresses the unique context for engaging Veterans as patients in VA health service research along with engaged VA clinical and operational leaders from the national healthcare system. In the model, we identify key domains and constructs critical to evaluating Veteran engagement and understanding the mechanisms of successful engagement. The discussion below considers the Veteran engagement model compared to other patient engagement frameworks, the unique value that it adds beyond other contemporary patient engagement models, and critical questions to address in evaluation and refinement of the model.

Unique Contributions of the Veteran Engagement Model

For over three decades, patient advocates, researchers, policy makers, and funders have moved toward engaging patients and other stakeholders as meaningful partners in health research. Early examples of stakeholder engagement occurred in community-based participatory research and community-engaged research.^20,21^ The VA HSR&D Conceptual Model Subgroup looked to these earlier engagement activities as examples of patient engagement in health research, yet found that the VA as an integrated health system presented new opportunities for patient and researcher co-design of health services, health care delivery, and implementation of models of care. Consequently, the group paid particular attention to articulating the processes needed for researchers, Veteran patients, and VHA health system stakeholders to build productive relationships. The focus on stakeholders, including Veterans, who have knowledge about healthcare delivery system differentiates the VA HSR&D patient engagement initiative from earlier community-engaged participatory research and the CTSA approaches.^22^ VA Veteran engagement advanced the idea that researchers needed to work closely with both Veterans and health system leaders in the VHA who could help them understand both patient perspectives and clinical and operational challenges and priorities at a deeper level to address gaps in healthcare delivery system through their research.

The processes described in the Veteran Engagement Model have similarity to the principles of engagement described by PCORI.^8,9^ However, the Veteran engagement model focused explicitly on the stakeholder group processes needed to create productive collaborative relationships, while other models focused on values. A 2019 patient engagement review reported that the most common foundational principles of engagement across studies were respect, equitable power, and trust.^23^ The VA model emphasizes interpersonal processes needed in research partnerships to create and maintain these sentiments potentally enhancing Veteran engagement, reducing tokenism, and increasing trust in research.^17,18,24^ While the Veteran Engagement Model does not specify measures of engagement, the articulation of important processes, influencing factors, and outcomes provides a preliminary logic model and basis for defining variables and measures for scientific study of Veteran engagement mechanisms and outcomes.^25^

The national VA HSR&D program has supported Veteran engagement in individual facilities and research projects since the development of the Veteran Engagement Model. National initiatives to facilitate engagement of Veterans in research leadership and individual research projects include the integration of Veterans on the national peer review panels and incentives for including Veterans’ feedback on applications for funding. Since the development of the model, individual researchers have integrated Veteran engagement in their research centers, programs, and projects.^26,27^ For example, the national Women’s Improvement Network within the VA HSR&D Women’s Health Research Network is comprised of women Veterans who are interested in advancing women’s health research in VA by bringing their research ideas to the forefront and guiding the efforts of researchers.^28^ Similarly, the VA EMPOWER QUERI provides an example of the use and adaptation of the Veteran engagement model. In this case, the model conceptualizes patient engagement as a continuum at each of three levels: direct care, organizational design and governance, and policy making^29^; the QUERI focuses on the direct care and organizational levels of engagement, examining how organizational capacity for innovation impacts implementation of care models designed to promote engagement and retention in care. The approach in this VA initiative is consistent with Grande et al.’s “information plus activation plus collaboration” category of patient engagement methods.^30^

Limitations, Strengths, and Future Research

Several important contemporary issues in Veteran engagement are not covered in this paper such as the engagement of underrepresented minorities in research, specific strategies used for engaging Veterans (e.g., Veterans’ Research Panels, engagement sessions), and the cost of Veteran engagement. Also, the model focuses on how Veteran engagement can be accomplished. From this perspective, the model anticipates processes that would produce successful engagement, facilitating factors rather than barriers, and positive outcomes. Future work is needed to consider a fuller logic model incorporating impeding processes, barriers, and unintended consequences of Veteran engagement in research.^31^ An important next step is to examine mediating and moderating factors of engagement and their relationships to Veteran engagement outcomes, especially research-to-policy and practice translation and benefits to Veterans in the VA healthcare system.^32^

## CONCLUSION

The model proposed in this article identifies the unique context of Veteran engagement in an integrated health system and health service research at the individual project and research center level that have engaged a wide range of other VA stakeholders. The value of the model is that it identifies constructs for understanding the mechanisms of Veteran engagement in research and its outcomes. It is intended for use by VA researchers planning Veteran engagement and to advance the transparency and impact of VA health service research.

## References

[1] Department of Veterans Affairs, National Center for Veterans Analysis and Statistics, Veteran Population. Website and webpage available at https://www.va.gov/vetdata/veteran_population.asp, April 2021. Accessed 8 Jan 2022.

[2] Haibach J, Hoerster K, Dorflinger L (2021). Research translation for military and veteran health: research, practice, policy. Translational Behav Med..

[3] Department of Veterans Affairs, Hays MT. A historical look at the establishment of the Department of Veterans Affairs Research and Development Program. Washington: Office of Research and Development, Veterans Health Administration, Department of Veterans Affairs 2010.

[4] Atkins D, Kupersmith J, Eisen S (2010). The Veterans Affairs experience: comparative effectiveness research in a large health system. Health Affairs..

[5] Kupersmith J, Eisen S (2012). A new approach to health services research. Arch Intern Med..

[6] Zickmund S, Knight S, Hamilton A, et al. Veteran engagement workgroup final report. Washington: Health Services Research and Development Service, Office of Research and Development, Veterans Health Administration, Department of Veterans Affairs 2015.

[7] Atkins D, Kilbourne AM, Shulkin D (2017). Moving from discovery to system-wide change: the role of research in a learning health care system: experience from three decades of health systems research in the Veterans Health Administration. Annu Rev Public Health..

[8] Frank L, Forsythe L, Ellis L (2015). Conceptual and practical foundations of patient engagement in research at the patient-centered outcomes research institute. Qual Life Res..

[9] Forsythe L, Heckert A, Margolis MK (2018). Methods and impact of engagement in research, from theory to practice and back again: early findings from the Patient-Centered Outcomes Research Institute. Qual Life Res..

[10] Kilbourne AM, Jones PL, Atkins D (2020). Accelerating implementation of research in learning health systems: lessons learned from VA Health Services Research and NCATS Clinical Science Tranlation Award programs. J Clin Transl Sci..

[11] Carr D, Howells A, Chang M, et al. An integrated approach to stakeholder engagement. Healthc Q. 2009;12 Spec No Ontario:62-70. 10.12927/hcq.2009.20754 19458512

[12] Nicolaidis C, Raymaker D, McDonald K (2011). Collaboration strategies in nontraditional community-based participatory research partnerships: lessons from an academic−community partnership with autistic self-advocates. Prog Community Health Partnersh..

[13] Baquet CR. A model for bidirectional community-academic engagement (CAE): overview of partnered research, capacity enhancement, systems transformation, and public trust in research. J Health Care Poor Underserved. 2012 Nov;23(4):1806-24. 11. 10.1353/hpu.2012.0155 PMC5393451 23698691

[14] Deverka PA, Lavallee DC, Desai PJ (2012). Stakeholder participation in comparative effectiveness research: defining a framework for effective engagement. J Comp Eff Res..

[15] Isler MR, Corbie-Smith G (2012). Practical steps to community engaged research: from inputs to outcomes. J Law Med Ethics..

[16] Carman KL, Dardess P, Maurer M (2013). Patient and family engagement: a framework for understanding the elements and developing interventions and policies. Health Aff (Millwood)..

[17] Shippee ND, Domecq Garces JP, Prutsky Lopez GJ (2015). Patient and service user engagement in research: a systematic review and synthesized framework. Health Expect..

[18] Domecq JP, Prutsky G, Elraiyah T (2014). Patient engagement in research: a systematic review. BMC Health Serv Res..

[19] Marlett N, Shklarov S, Marshall D (2015). Building new roles and relationships in research: a model of patient engagement research. Qual Life Res.

[20] Blumenthal DS (2011). Is community-based participatory research possible?. Am J Prev Med..

[21] Leung MW, Yen IH, Minkler M (2004). Community based participatory research: a promising approach for increasing epidmiology’s relevance in the 21^st^ century. Int J Epidemiol..

[22] Jull J, Giles A, Graham ID (2017). Community-based participatory research and integrated knowledge translation: advancing the co-creation of knowledge. Implement Sci..

[23] Harrison JD, Auerbach AD, Anderson W (2019). Patient stakeholder engagement in research: a narrative review to describe foundational principles and best practice activities. Health Expect..

[24] Concannon TW, Fuster M, Saunders T (2014). A systematic review of stakeholder engagement in comparative effectiveness and patient-centered outcomes research. J Gen Intern Med..

[25] Luger TM, Hamilton AB, True G (2020). Measuring community-engaged research contexts, processes, and outcomes: a mapping review. Milbank Q..

[26] Brys NA, Whittle J, Safdar N (2018). Development of a veteran engagement toolkit for researchers. J Comp Eff Res..

[27] Hyde J, Wendleton L, Fehling K, et al. Strengthening Excellence in Research through Veteran Engagement (SERVE): toolkit for veteran engagement in research (Version 1, 2018) Available: https://www.hsrd.research.va.gov/for_researchers/serve/, March 2019. Accessed 8 Jan 2022.

[28] Frayne S, Hamilton A, Yano E. VA Women’s Health Research Network: accelerating research impacts and advancing learning healthcare system principles. VA HSR&D National Cyberseminar Available: https://www.hsrd.research.va.gov/for_researchers/cyber_seminars/archives/video_archive.cfm?SessionID=3562, November 2018. Accessed 8 Jan 2022.

[29] Hamilton A, Farmer M, Moin T (2017). Enhancing mental and physical health of women through engagement and retention (EMPOWER): a protocol for a program of research. Implement Sci..

[30] Grande SW, Faber MJ, Durand MA, Thompson R, Elwyn G (2014). A Classification model of patient engagement methods and assessment of their feasibility in real-world settings. Patient Education and Counseling..

[31] Bowen DJ, Hyams T, Goodman M (2017). Systematic review of quantitative measures of stakeholder engagement. Clin Transl Sci..

[32] Wells KB, Jones L, Chung B (2013). Community-partnered cluster-randomized comparative effectiveness trial of community engagement and planning or resources for services to address depression disparities. J Gen Intern Med..

